# Eating or Feeding Our Young: A Response to Recent Commentaries

**DOI:** 10.15171/ijhpm.2017.136

**Published:** 2017-11-26

**Authors:** Terrence Sullivan, Vidhi Thakkar

**Affiliations:** Institute of Health Policy Management and Evaluation, University of Toronto, Toronto, ON, Canada.

We are very grateful for the three thoughtful commentaries from our respective colleagues, Hunter and Frank,^1^ Barer and Bryan,^2^ as well as Adams^3^ in this volume of *IJHPM*. We respond to their comments in turn.

Our original commentary^4^ was intended to open a discussion on public spending on Health Services and Policy Research (HSPR), motivated in part by how little money was spent on HSPR as a fraction of total health research spending in Canada (about 8%).^5^ In addition, a fact to which the first author can attest as former chair of a CIHR HSPR review panel, the fraction of HSPR proposals recommended for funding has been poor relative to basic and clinical science. This has led to the suggestions from younger applicants that the mature HSPR community is predisposed to eat their young.

Hunter and Frank made the clear point that there is a significant amount of money flowing to investigators salaries and overhead in the United States as well as to a lesser extent in the United Kingdom while Canada remains the more parsimonious with a focus on direct research costs and some career support to young and senior investigators. Hunter and Frank note that direct ‘academic’ research, without following up the direct implications for clinical, institutional and public policy decision-making often winds up being stockpiled without much effect.

In the Canadian context, a new cadre of ‘embedded researchers’ who work outside of the university in delivery institutions and clinical settings as well as management settings is being established. This approach may help to bridge the gap between purely academic pursuit and change projects, which may have direct benefits within health service delivery environments and health policy shops within government – a move which would speak to the comments from Barer and Bryan as well for stronger research-decision making cooperative efforts. A new training curriculum is being advanced and more applied fellowships in delivery organizations are being supported to ensure that doctoral and post-doctoral trainees are building the skills to successfully engage with decision-makers at multiple levels in the healthcare delivery systems.^6^

All three commentators point to the old saw that HSPR research needs to work with decision makers to advance research with impact. This requires more than interaction or ‘talk.’ It requires adapting the original research plan to accommodate the sometimes-messy requirements and contexts for decision makers. For these many reasons, it is desirable to rejig training programs and venues to allow HSPR researchers to get grounding in the delivery system realities and field research as an essential to create impact. Different field contexts remain a central challenge in dealing with the complex issues of advancing improvement and change in real world settings.^7^

Barer and Bryan expressed some skepticism about the ranking of the public spending and that there appears to be little relation between the investment and outcome – and fair enough. We agree that the United Kingdom does deliver better *health sector* performance, pound for pound – so to speak – than the other two delivery systems. But the exact link between the money spent and the health *outcomes* achieved is unlikely to be a simple, uniform or linear to be sure. Outcomes will depend on what is being measured. Neither life expectancy nor cancer survival would meet the test of better outcomes for the United Kingdom for example. They reference the Strategy for Patient Oriented Research (SPOR) in Canada as a step toward embedding research more directly in the delivery system. We agree. The aforementioned new flight of Health System Impact Awards is also well designed to place researchers directly into novel learning environments in the health system and health sector organizations, with tailored improvement goals and project.^8^

There is nothing ‘magical’ about trying to estimate what measure of public money spent on HSPR would achieve some basic scale of effort and spending impact. Public money is certainly only a part of the story and the single source we could safely estimate. The scale of philanthropic contributions to HSPR are especially visible in the United Kingdom and the United States. Additionally, there are many consultants working in this terrain, as Adams notes in his commentary. The Kings Fund and the Nuffield Trust, among others in the United Kingdom have had meaningful influence on policy makers and contribute to reform through interactions with decision makers. The Commonwealth Fund and Robert Wood Johnson Foundations in the United States – to name just two of the many large American philanthropic enterprises – have had significant impacts on decision making and training in the United States and beyond.

Building new capacity to bridge research and practice change will cost money outside of the academic environment and will engage individuals with applied research aspiration to take on organizational remapping and improvement iterations on a going forward basis. It is a bit nihilist to suggest the money does not matter. It matters indeed if the money is training investigators who are intentionally trained and channeled to make their way and generate improvement impact in health sector delivery systems and the corridors of public decision making. It matters if new investigators favor methodologically guided reform rather than describing interesting problems and theoretical challenges in a less real and very academic world where few people read their work.

Our commentators note that the conduct of HSPR has traditionally not been well-linked to reform as if there are two solitudes. With the modernization of the training program, the field is evolving in a new way. It does seem to us that such solitudes are less common as HSPR researchers are more actively involved inside delivery sector organizations and policy environments where they face challenges on matters of pragmatic policy and politics.^8^

It does appear that bridging meetings between the solitudes are far more common than ever before. At this stage of things policy makers and health services researchers do look for better ways together to build bridges to advance reform.^9^

In conclusion, we are pleased to see the spectrum of response to modest proposals. Having no money or a fixed or shrinking fraction of research funds is not likely to increase health systems impact. Likewise, it is our view that reasonable people working together can build common nomenclature for cross-jurisdictional HSPR research to instrument comparative HSPR work. The alternative seems to be some an ill-defined collection of HSPR research domains without common meaning and metrics that bind the field together. In our view, we should feed the HSPR young with money, health systems impact opportunities, and comparative knowledge.

## Acknowledgement

We would like to acknowledge Meghan McMahon from the Institute of Health Services and Policy Research for information on the Health Systems Impact Fellowship.

## Ethical issues

Not applicable.

## Competing interests

Authors declare that they have no competing interests.

## Authors’ contributions

Both authors contributed equally to the writing and editing of this manuscript. This was completed as a part of a Senior Fellows Learning Activity at the Institute of Health Policy Management and Evaluation, University of Toronto, Toronto, ON, Canada.
